# Sleep Quality, Sleep Duration, and the Risk of Adverse Clinical Outcomes in Patients With Myocardial Infarction With Non-obstructive Coronary Arteries

**DOI:** 10.3389/fcvm.2022.834169

**Published:** 2022-02-28

**Authors:** Chun-Yan Zhu, Hui-Lin Hu, Guan-Min Tang, Jing-Chao Sun, Hui-Xiu Zheng, Chang-Lin Zhai, Chao-Jie He

**Affiliations:** ^1^Department of Anesthesiology, The Affiliated Hospital of Jiaxing University, Jiaxing, China; ^2^Department of Cardiology, The Affiliated Hospital of Jiaxing University, Jiaxing, China

**Keywords:** MINOCA, sleep quality, sleep duration, all-cause mortality, MACE

## Abstract

**Background:**

Myocardial infarction with non-obstructive coronary arteries (MINOCA) is a heterogeneous entity with varying underlying etiologies and occurs in ~5–10% of patients with acute myocardial infarction. Sleep disorders and short sleep duration are common phenomena experienced by patients with coronary heart disease and are associated with poor clinical outcomes. However, the association between sleep quality, sleep duration, and the MINOCA prognosis is less clear.

**Methods:**

We performed a prospective observational study of 607 patients with MINOCA between February 2016 and June 2018. The mean follow-up period was 3.9 years. Sleep quality and sleep duration were measured by the Chinese version of the Pittsburgh Sleep Quality Index. The primary endpoint was all-cause mortality, and the secondary endpoint was major adverse cardiovascular events (MACE), defined as a composite of cardiovascular death, non-fatal myocardial infarction, stroke and heart failure hospitalization.

**Results:**

During the follow-up period, all-cause death occurred in 69 participants and 105 participants developed MACE. The Kaplan–Meier survival analysis demonstrated a significant association between poor sleep quality and all-cause mortality (log-rank *P* = 0.005) and MACE (log-rank *P* = 0.004). Multivariable Cox regression model indicated that poor sleep quality was an independent predictor of all-cause mortality as well as MACE [adjusted hazard ratio (HR) = 1.649; 95% confidence interval (CI), 1.124–2.790; *P* < 0.001; and adjusted HR = 1.432; 95% CI, 1.043–2.004; *P* = 0.003, respectively]. For sleep duration, short sleep duration (<6 h/d) was significantly associated with an increased risk of all-cause mortality and MACE (adjusted HR = 1.326; 95% CI, 1.103–1.812; *P* = 0.004; and adjusted HR = 1.443; 95% CI, 1.145–1.877; *P* < 0.001, respectively), whereas long sleep duration was not (>8 h/d). A poorer sleep profile (including poor sleep quality and short sleep duration) was associated with a 149.4% increased risk of death (HR = 2.494; 95% CI, 1.754–4.562; *P* < 0.001) and a 96.7% increased risk of MACE (HR = 1.967; 95% CI, 1.442–3.639; *P* < 0.001) than those with neither.

**Conclusion:**

Sleep disorders were common among Chinese patients with MINOCA. Poor sleep quality and short sleep duration were independently associated with an increased risk of all-cause mortality and MACE in the MINOCA population. Meanwhile, a poor sleep profile has an additive effect with regard to cardiovascular risks; in these populations, efforts should be made to improve both sleep quality and sleep duration for secondary cardiovascular prevention.

**Clinical Trial Registration:**

http://www.chictr.org.cn, identifier: ChiCTR2000040701.

## Introduction

Myocardial infarction with non-obstructive coronary arteries (MINOCA) is a working diagnosis characterized by the acute presentation of myocardial infarction with no significant stenosis of the coronary arteries after angiography (stenosis <50%). MINOCA accounts for approximately 5%−10% of all patients with acute myocardial infarction (AMI) (1–3). The underlying pathophysiological mechanisms of MINOCA are varied and may include plaque rupture or ulceration, epicardial coronary spasm, coronary microvascular dysfunction, thromboembolism, spontaneous coronary artery dissection, and supply–demand mismatch. MINOCA should not be a definitive diagnosis, but an umbrella term designates a heterogeneous group of conditions (4–6). The diagnosis and management of MINOCA are still challenging in clinical practice and require further evaluation (7).

Sleep is a complex physiological process that comprises approximately one-third of human life. A comprehensive sleep profile consists of both sleep quality and sleep duration (8). Sleep disorders, including insomnia, insufficient sleep duration, and poor sleep quality, affect >45% of the global population and have become a crucial public health concern (9). Accumulating evidence has demonstrated that sleep impairment is a considerable risk factor for stroke, coronary artery disease (CAD), heart failure, and cardiovascular death (10–13). In addition, earlier studies also revealed a significant association between poor sleep quality and short sleep duration with higher rates of all-cause mortality in the general population (14). A recent study comprising patients with acute coronary syndrome (ACS) showed that individuals suffer from more sleep disturbances compared with those without and have increased morbidity and impaired quality of life (15). Despite the high prevalence of sleep impairment and worsened clinical prognosis in patients with CAD, previous research on the population with MINOCA has been scarce.

MINOCA has been recognized since the early 1980's, but only recently have diagnostic criteria and medical management been recommended (4, 16). It should not be assumed that prognostic factors proven in CAD patients are equivalent to those in the MINOCA population. To date, there are no published data on the prevalence of sleep disorders in patients with MINOCA, and the association between sleep impairment and clinical outcomes has not been determined. In the present study, we aimed to examine the prevalence of sleep disorders and the association between sleep quality, sleep duration, and poor prognosis in patients with MINOCA. In addition, we also investigated the joint effects of sleep quality and sleep duration on adverse outcomes. To minimize the potential impact of confounders, we collected additional baseline characteristics, including anxiety, depression, and obstructive sleep apnea–hypopnea syndrome (OSAHS) (17–19).

## Methods

### Study Population and Design

We consecutively recruited 607 patients with a clinical presentation of MINOCA who were admitted to the Affiliated Hospital of Jiaxing University from February 2016 to June 2018. MINOCA was defined according to the European Society of Cardiology working group position paper with some modifications: (1) a definite diagnosis of AMI based on the Fourth Universal Definition of Myocardial Infarction, (2) coronary angiography demonstrating no obstructive coronary artery (<50% stenosis) in any major epicardial vessel, and (3) no other specific alternate diagnosis for the acute presentation (5, 20). The following exclusion criteria were feasible in this pragmatic trial: (1) the diagnosis of MINOCA was closed to the clinical algorithm as recommended by the American Heart Association; that is, efforts were made to investigate the underlying cause for the myocardial injury, mimic myocardial infarction as follows but not limited to sepsis, pulmonary embolism, myocardial contusion, overlooked obstructive CAD, Takotsubo syndrome, and myocarditis; (2) age <18 years; (3) patients with dementia or cognitive impairment; (4) patients with a terminal illness with a life expectancy of <1 year; and (5) refusal to participate in the study.

This study protocol was approved by the Ethics and Research Committee of the Affiliated Hospital of Jiaxing University. All participants provided written informed consent before enrollment. The trial was registered at the Chinese Clinical Trial Registry (ChiCTR; http://www.chictr.org.cn; ChiCTR2000040701).

### Data Collection

We used the HaiTai (version 3.0) inpatient system to collect clinical information, including demographics; laboratory data; electrocardiogram, echocardiography, and angiographic findings; psychiatric conditions; and discharge medications. All patients were followed up with half-yearly intervals scheduled through the outpatient clinic visit, telephone interview, or instant messaging application (WeChat) after discharge. Data collectors were blinded to the group assignment during both baseline and endpoint assessments. Due to the lack of definitive treatment guidelines for MINOCA, renin-angiotensin-aldosterone system (RAAS) inhibitors, β-blockers, statins, and antiplatelet therapies were prescribed by the physician's discretion.

### Sleep Assessment

All participants were requested to complete the Pittsburgh Sleep Quality Index (PSQI) for the subjective assessment of sleep quality within the past 30 days. The Chinese version of the PSQI comprises 19 self-rated questions grouped into seven domains and five questions rated by the bed partner or roommate (21). The seven components include subjective quality of sleep, sleep latency, sleep duration, habitual sleep efficiency, sleep disturbances, use of sleeping pills, and diurnal dysfunction. Each component score is weighed equally from 0 to 3, with a whole PSQI score ranging from 0 to 21. The PSQI has been demonstrated to have high internal consistency (0.85), test–retest reliability (0.83), and construct validity in the Chinese population (21). Sleep quality as measured by PSQI was categorized dichotomously as good sleep quality (PSQI ≤ 5) or poor sleep quality (PSQI > 5), with a diagnostic sensitivity of 90% and specificity of 87% (22). For sleep duration, subjects were asked, “How many hours do you usually sleep per 24 h?” with the following three answer options: <6 h, 6–8 h, and >8 h. Physicians who were blinded to the study design conducted the questionnaire survey. For illiterate patients, the researcher read the questions and filled out the scale for them.

### Primary and Secondary Endpoints

The primary study endpoint was all-cause mortality, and the secondary endpoint was the first occurrence of major adverse cardiovascular events (MACE) during follow-up. MACE was defined as a composite of cardiovascular death, non-fatal myocardial infarction, stroke and heart failure hospitalization. Cardiovascular death was defined as death only with clinical evidence of ACS, severe cardiac arrhythmia, or refractory congestive heart failure. Two senior cardiologists without any knowledge of the participant allocation confirmed the endpoints. Discrepancies were adjudicated by a third researcher.

### Statistical Analysis

Continuous variables were presented as mean ± standard deviation or median with interquartile range and categorical variables as counts or proportions (%). Intergroup differences were analyzed using the Student's *t*-test or Mann–Whitney *U*-test for continuous data and the chi-square test or Fisher's exact test for categorical data, as appropriate. Multiple group comparisons were carried out by one-way analysis of variance. The cumulative event-free rates comparing participants with or without sleep disorders were estimated using the Kaplan–Meier method, and differences between groups were examined by the log-rank test.

The multivariate Cox proportional hazards models were applied to evaluate the relationship between sleep quality, sleep duration, and clinical outcomes. Hazard ratios (HRs) and 95% confidence interval (CIs) were calculated using Cox proportional hazards regression. The appropriateness of the model was checked graphically using log-minus-log plots and found to be suitable. We identified possible covariates to be retained in the analyses through a stepwise selection procedure with an entrance and stay criteria of *P* < 0.10, forcing the known risk and protective factors into all models a priori. Therefore, variables entered into the multivariable model for death events included depression, anxiety, OSHAS, RAAS inhibitors, and statins. After the model was created, the remaining candidate predictors (i.e., smoking, diabetes, and β-blockers) were retested individually with a sensitivity analysis to determine their influence on effect estimates. Using a similar pattern, we constructed multivariate models separately for all-cause mortality and MACE. All statistical analyses were performed in accordance with intention-to-treat approaches using SPSS software (SPSS 23.0, SPSS Inc., Chicago, IL). Two-sided *P* < 0.05 was considered statistically significant.

## Results

### Comparison of Patient Characteristics

**Figure 1:**
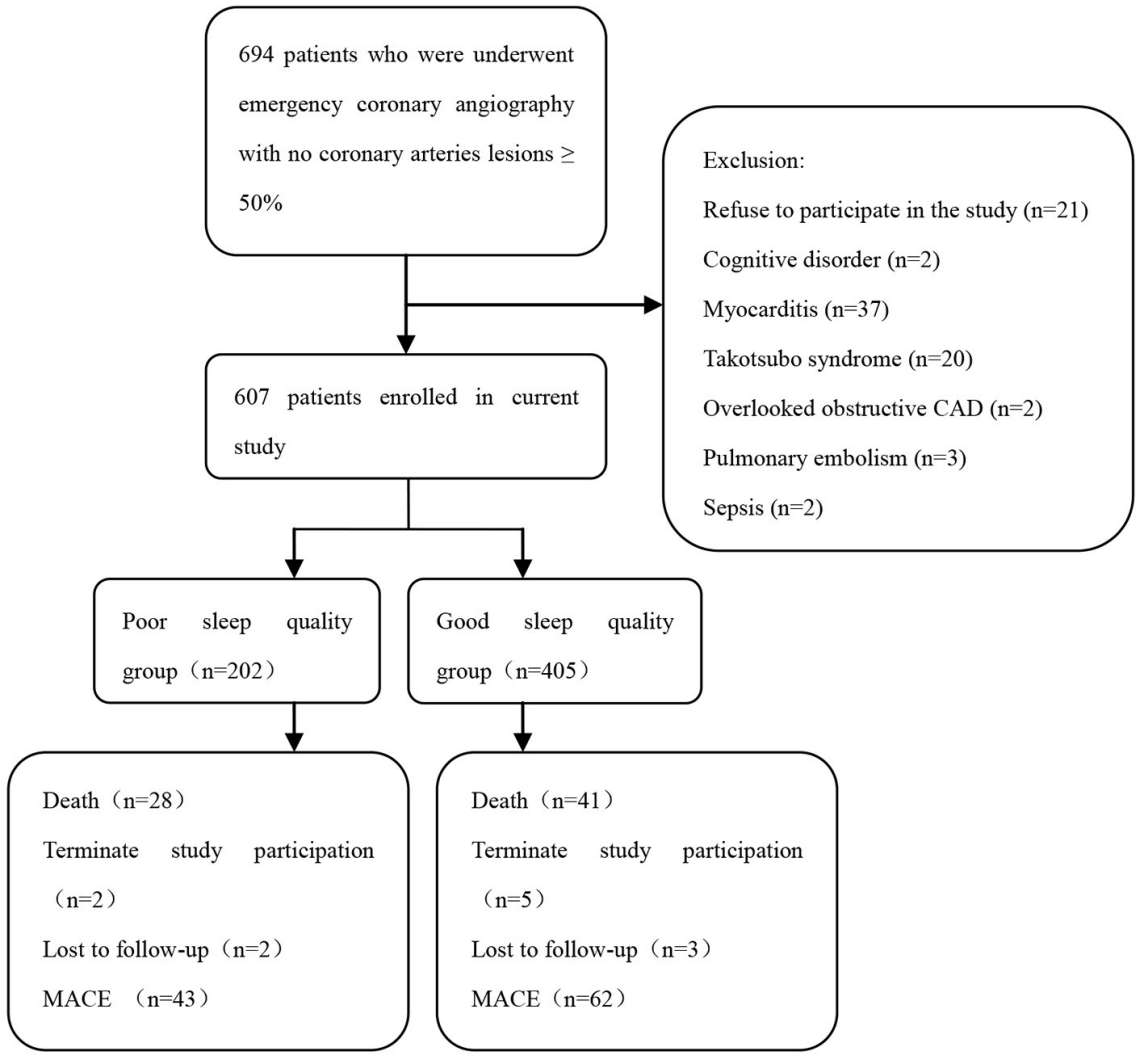
Flowchart of the selection process and dropouts of current study.

A total of 694 patients with myocardial infarction underwent emergency angiography that revealed no significant coronary stenosis, of whom 607 (87.5%) individuals met the diagnostic criteria for MINOCA. Eighty-seven patients were excluded from the present study: 2 with cognitive disorder, 37 with myocarditis, 20 with Takotsubo syndrome, 2 with overlooked obstructive CAD, 2 with sepsis, 3 with pulmonary embolism, and 21 patients who refused to participate in the study (Figure 1).

Tables 1, 2 summarize the baseline characteristics of the study population stratified by sleep quality and sleep duration. The mean baseline PSQI was 6.7 ± 4.4 (range, 0–21), with poor sleep quality (PSQI > 5) in 202 participants. The incidence of poor sleep quality was 33.3% in the Chinese MINOCA population. The mean reported sleep duration was 7.3 ± 1.4 h, with 11.9% of individuals reporting that they typically slept for fewer than 6 h each day. Patients with poor sleep quality tended to be older, smokers, and more likely to have a higher prevalence of stroke and hypertension (Table 1). Those individuals with short sleep duration (<6 h/d) tended to have a higher body mass index and were more likely to have a history of stroke (Table 2). All groups had similar rates of angiographic findings and receipt of evidence-based medications at discharge. Only 28 patients (13.9%) with poor sleep quality agreed to take sleeping pills with alprazolam or estazolam.

**Table 1:** Demographic and clinical characteristics of the study population with poor sleep quality and good sleep quality.

**Characteristics**	**Poor sleep quality PSQI > 5 (*n* = 202)**	**Good sleep quality PSQI ≤5 (*n* = 405)**	***P*-value**
**Demographics**			
Age, mean ± SD, y	63.9 ± 12.7	62.4 ± 12.6	0.039
Female, *n* (%)	115 (56.9)	238 (58.8)	0.452
BMI, mean ± SD, kg/m^2^	24.8 ± 3.7	24.4 ± 3.6	0.171
**Risk factors**, ***n*** **(%)**			
Smoking,	53 (26.2)	91 (22.5)	0.023
Hypertension	112 (55.4)	200 (49.4)	0.007
Diabetes	41 (20.3)	85 (21.0)	0.496
Hyperlipemia	48 (23.8)	101 (24.9)	0.639
Stroke history	10 (5.0)	14 (3.5)	0.043
Heart failure	7 (3.5)	16 (4.0)	0.231
**Medications at discharge**, ***n*** **(%)**			
Anti-platelets	172 (85.1)	351 (86.7)	0.426
βBlockers	119 (58.9)	244 (60.2)	0.756
RAAS inhibitors	98 (48.5)	200 (49.4)	0.361
Statins	170 (84.2)	350 (86.4)	0.483
**Electrocardiographic changes on admission**, ***n*** **(%)**			
STEMI	25 (12.4)	53 (13.1)	0.452
NSTEMI	177(87.6)	352 (86.9)	0.452
**Echocardiography, mean** **±SD**
LVEF (%)	54.2 ± 13.2	54.5 ± 13.4	0.747
**Laboratory parameters on admission, mean** **±SD**			
Pro-BNP (pg/mL)	878.9 ± 1,275.2	930.1 ± 1,322.5	0.457
cTnT (ng/mL)	2.3 ± 1.1	2.4 ± 1.1	0.245
CRP (mg/L)	15.9 ± 4.8	16.3 ± 5.2	0.812
**Angiographic data**, ***n*** **(%)**			
Normal vessels	20 (9.9)	43 (10.6)	0.345
Stenosis ≤ 30%	96 (47.5)	202 (49.9)	0.345
30% < Stenosis <50%	86 (42.6)	160 (39.5)	0.345

*BMI, body mass index; RAAS, rennin-angiotensin-aldosterone system; STEMI, ST-segment elevation myocardial infarction; NSTEMI, non-ST-segment elevation myocardial infarction; LVEF, left ventricular ejection fraction; Pro-BNP, pro-brain natriuretic peptide; cTnT, cardiac troponin T; CRP, C reactive protein*.

**Table 2:** Demographic and clinical characteristics of the patients classified by level of sleep duration.

**Characteristics**	** <6 h (*n* = 72)**	**6–8 h (*n* = 430)**	**>8 h (*n* = 105)**	***P*-value**
**Demographics**				
Age, mean ± SD, y	63.2 ± 12.9	62.8 ± 12.6	62.3 ± 12.7	0.523
Female, *n* (%)	45(62.5)	245 (57.0)	63 (60.0)	0.122
BMI, mean ± SD, kg/m^2^	25.2 ± 3.6	24.3 ± 3.6	24.6 ± 3.7	0.038
**Risk factors**, ***n*** **(%)**				
Smoking	17 (23.6)	95(22.1)	25 (23.8)	0.637
Hypertension	34 (47.2)	210 (48.8)	49 (46.7)	0.899
Diabetes	15 (20.8)	91 (21.2)	20 (19.0)	0.232
Hyperlipemia	18 (25.0)	105 (24.4)	26 (24.8)	0.636
Stroke history	6 (8.3)	13 (3.0)	5 (4.8)	0.012
Heart failure	3 (4.2)	20 (4.7)	3 (2.9)	0.065
**Medications at discharge**, ***n*** **(%)**				
Anti-platelets	62 (86.1)	369 (85.8)	92 (87.6)	0.633
β Blockers	42 (58.3)	258 (60.0)	63 (60.0)	0.323
RAAS inhibitors	35 (48.6)	211 (49.1)	52 (49.5)	0.332
Statins	62 (86.1)	369 (85.8)	89 (84.8)	0.258
**Electrocardiographic changes on admission**, ***n*** **(%)**				
STEMI	10 (13.9)	55 (12.8)	13 (12.4)	0.745
NSTEMI	62 (86.1)	375 (87.2)	92 (87.6)	0.745
**Echocardiography, mean** **±SD**				
LVEF (%)	54.6 ± 13.6	54.3 ± 13.4	54.0 ± 13.3	0.223
**Laboratory parameters on admission, mean** **±SD**				
Pro-BNP (pg/mL)	942.9 ± 1,296.3	926.1 ± 1,320.3	853.1 ± 1,295.3	0.363
cTnT (ng/mL)	2.3 ± 1.1	2.4 ± 1.1	2.3 ± 1.0	0.699
CRP (mg/L)	15.9 ± 4.9	16.2 ± 5.2	16.4 ± 5.4	0.321
**Angiographic data**, ***n*** **(%)**				
Normal vessels	8 (11.1)	43 (10.0)	12 (11.4)	0.553
Stenosis ≤ 30%	35 (48.6)	211 (49.1)	52 (49.5)	0.553
30% < Stenosis <50%	29 (40.3)	176 (40.9)	41 (39.0)	0.553

*BMI, body mass index; RAAS, rennin-angiotensin-aldosterone system; STEMI, ST-segment elevation myocardial infarction; NSTEMI, non-ST-segment elevation myocardial infarction; LVEF, left ventricular ejection fraction; Pro-BNP, pro-brain natriuretic peptide; cTnT, cardiac troponin T; CRP, C reactive protein*.

### Clinical Outcomes

A total of 595 were successfully followed up until death or the end of the trial, and only five participants were lost to follow-up. After a mean follow-up period of 3.9 years, a total of 69 deaths and 105 MACE had occurred. Of these, there were 28 deaths (13 cardiovascular deaths, 3 cancer deaths, 1 hemorrhage-related deaths, 1 death due to leukemia, 10 unexplained deaths) and 43 MACE (13 cardiovascular deaths, 15 non-fatal myocardial infarctions, 1 stroke and 14 heart failure hospitalizations) in patients with poor sleep quality; in the control group, there were 41 deaths (19 cardiovascular deaths, 7 cancer deaths, 1 death from accident, 2 hemorrhage-related deaths, 2 death from infectious disease, 10 unexplained deaths) and 62 MACE (19 cardiovascular deaths, 22 nonfatal myocardial infarctions, 3 strokes and 18 heart failure hospitalizations).

We categorized sleep duration into three categories: short (<6 h), average (6–8 h), and long (>8 h). Table 3 presents the detailed causes of MACE in each group.

**Table 3:** Major clinical outcomes of the study population during follow-up.

**Primary and secondary endpoints**	** <6 h (*n* = 72)**	**6–8 h (*n* = 430)**	**>8 h (*n* = 105)**	***P*-value**
All-cause mortality, *n* (%)	13 (18.1)	44 (10.2)	12 (11.4)	0.012
Cardiovascular death	6 (8.3)	22 (5.1)	4 (3.8)	<0.001
Cancer	2 (2.8)	4 (1.0)	4 (3.8)	0.072
Other	1 (1.4)	5 (1.2)	1 (1.0)	0.530
Unexplained	4 (5.6)	13 (3.0)	3 (2.9)	0.156
MACE				
Cardiovascular death	5 (6.9)	20 (4.7)	7 (6.7)	0.039
Non-fatal myocardial infarction	9 (12.5)	24 (5.6)	4 (3.8)	0.003
Stroke	1 (1.4)	2 (0.5)	1 (0.9)	0.755
Heart failure hospitalization	7 (9.7)	20 (4.7)	5 (4.8)	0.044

### Association Between Sleep Quality and Sleep Duration With Clinical Outcomes

**Figure 2:**
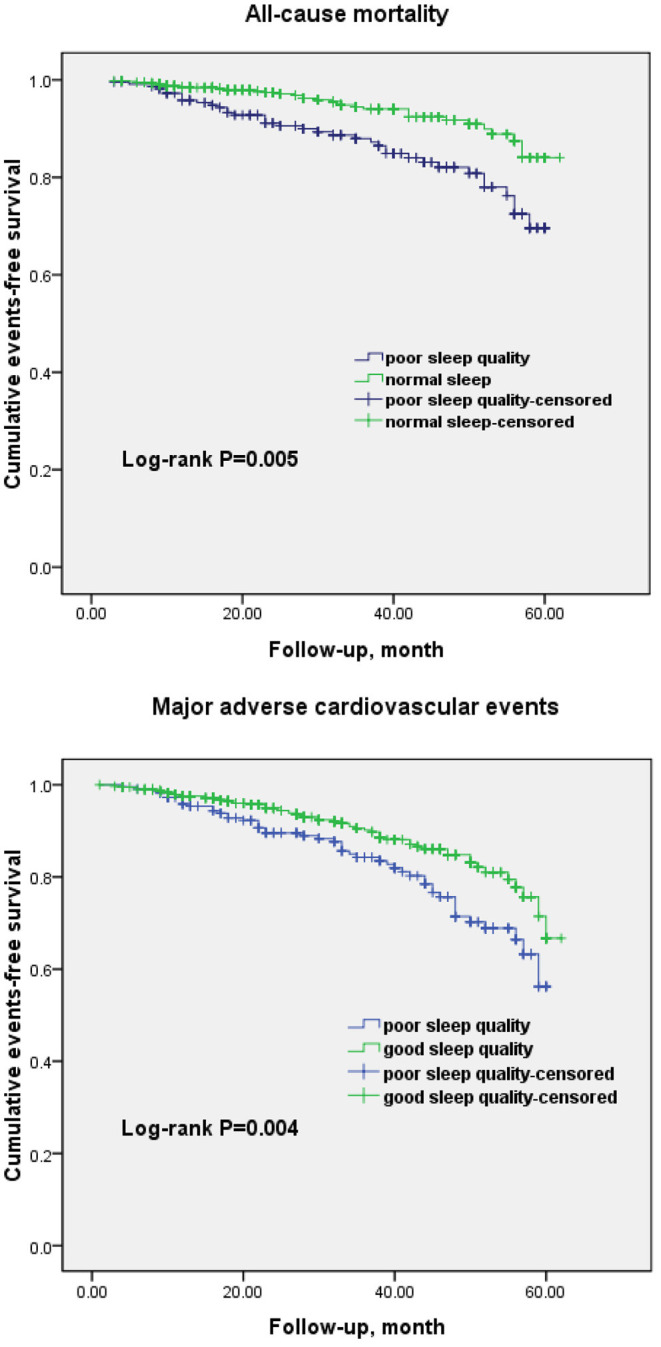
Kaplan–Meier curves for all-cause mortality and major adverse cardiovascular events in patients with poor sleep quality and good sleep quality.

The Kaplan–Meier cumulative survival analysis demonstrated a significant association between poor sleep quality and all-cause mortality (log-rank *P* = 0.005) and MACE (log-rank *P* = 0.004; Figure 2). Univariate Cox regression analysis showed that patients who reported poor sleep quality had an 85.6% higher risk of death (HR = 1.856; 95% CI, 1.263–2.992; *P* = 0.002) and a 66.5% higher risk of MACE (HR = 1.665; 95% CI, 1.131–2.237; *P* = 0.002) than those who had good sleep quality (Table 4). After adjusting for the known risk factors and potential protective medications in the multivariate analysis, poor sleep quality remained an independent predictor of all-cause mortality as well as MACE in the MINOCA population (adjusted HR = 1.649; 95% CI, 1.124–2.790; *P* < 0.001; and adjusted HR = 1.432; 95% CI, 1.043–2.004; *P* = 0.003, respectively; Table 5).

**Table 4:** Univariate Cox regression of variables influencing all-cause mortality and MACE.

**Variables**	**All-cause mortality**		**MACE**	
	**HR (95% CI)**	***P*-value**	**HR (95% CI)**	***P*-value**
Age (per decade increase)	1.012 (0.956–1.069)	0.599	1.022 (0.923–1.082)	0.756
Female	0.872 (0.643–1.157)	0.335	0.889 (0.770–1.157)	0.344
Smoking	1.269 (0.831–1.533)	0.219	1.120 (0.879–1.337)	0.701
Hypertension	1.101 (0.791–1.335)	0.870	1.166 (0.854–1.611)	0.561
Diabetes	1.149 (0.839–1.372)	0.521	1.112 (0.899–1.545)	0.419
Heart failure	1.312 (0.918–1.990)	0.087	1.393 (0.896–2.063)	0.102
Anti-platelets	0.901 (0.755–1.224)	0.671	0.786 (0.622–1.118)	0.322
β-Blockers	0.789 (0.579–1.424)	0.241	0.651 (0.502–1.213)	0.250
RAAS inhibitors	0.696 (0.410–0.989)	0.035	0.611 (0.422–0.948)	<0.01
Statins	0.423 (0.268–0.639)	<0.001	0.523 (0.223–0.639)	<0.001
STEMI	6.233 (2.877–17.441)	<0.001	7.676 (2.656–17.434)	<0.001
Depression	5.113 (3.221–8.442)	<0.001	3.019 (2.042–3.911)	<0.001
Anxiety	1.523 (1.004–2.233)	0.027	1.412 (1.023–2.023)	0.022
OSAHS	1.732 (1.235–2.795)	0.003	1.921 (1.444–3.221)	<0.001
Poor sleep quality	1.856 (1.263–2.992)	<0.001	1.665 (1.131–2.237)	0.002

*MACE, major adverse cardiovascular events; HR, hazard ratio; RAAS, rennin-angiotensin-aldosterone system; STEMI, ST-segment elevation myocardial infarction; OSAHS, obstructive sleep apnea-hypopnea syndrome*.

**Table 5:** Multivariate Cox regression of variables influencing all-cause mortality and MACE.

**Variables**	**All-cause mortality**		**MACE**	
	**HR (95% CI)**	***P*-value**	**HR (95% CI)**	***P*-value**
Heart failure	1.263 (0.911–1.893)	0.089	NA	NA
RAAS inhibitors	0.681 (0.414–0.979)	0.035	0.633 (0.421–0.921)	<0.01
Statins	0.409 (0.261–0.599)	<0.001	0.413 (0.278–0.598)	<0.001
STEMI	5.754 (2.457–15.314)	<0.001	7.123 (2.199–16.454)	<0.001
Depression	5.791 (3.561–9.892)	<0.001	2.972 (2.001–3.987)	<0.001
Anxiety	1.563 (1.006–2.374)	0.025	1.442 (1.047–2.014)	0.024
OSAHS	1.512 (1.121–2.345)	0.032	1.632 (1.211–2.785)	0.002
Poor sleep quality	1.649 (1.124–2.790)	<0.001	1.432 (1.043–2.004)	0.003

*MACE, major adverse cardiovascular events; HR, hazard ratio; NA, not applicable; RAAS, rennin-angiotensin-aldosterone system; STEMI, ST-segment elevation myocardial infarction; OSAHS, obstructive sleep apnea-hypopnea syndrome*.

For sleep duration, individuals in the group who slept fewer than 6 h per day had a significantly increased risk of all-cause mortality and MACE (adjusted HR = 1.326; 95% CI, 1.103–1.812; *P* = 0.004; and adjusted HR = 1.443; 95% CI, 1.145–1.877; *P* < 0.001, respectively). However, individuals with a long sleep duration (>8 h/d) did not have a statistically significant increased risk (adjusted HR = 1.081; 95% CI, 0.921–1.178; *P* = 0.125; and adjusted HR = 1.102; 95% CI, 0.949–1.204; *P* = 0.095, respectively; Table 6).

**Table 6:** Relationship of sleep duration with all-cause mortality and MACE during follow up period.

**Sleep duration**	**All-cause mortality**		**MACE**	
	**HR (95% CI)**	***P*-value**	**HR (95% CI)**	***P*-value**
**Categorical**				
<6 h	1.326 (1.103–1.812)	0.004	1.443 (1.145–1.877)	<0.001
6–8 h	1.0	-	1.0	-
>8 h	1.081 (0.921–1.178)	0.125	1.102 (0.949–1.204)	0.095

*MACE, major adverse cardiovascular events; HR, hazard ratio*.

When taking into consideration the joint effects of sleep quality and sleep duration, individuals with a poorer sleep profile (including poor sleep quality and short sleep duration) had a 149.4% increased risk of death (HR = 2.494; 95% CI, 1.754–4.562; *P* < 0.001) and a 96.7% increased risk of MACE (HR = 1.967; 95% CI, 1.442–3.639; *P* < 0.001) than those with neither.

## Discussion

In this prospective observational cohort study, patients were admitted following a clinical presentation with MINOCA and followed for almost 4 years (mean 3.9 years, maximum 5 years). We observed a high prevalence of sleep disorders among MINOCA patients. Meanwhile, in those populations, both poor sleep quality and short sleep duration were independently associated with a higher risk of cardiovascular events and death. Furthermore, a poorer sleep profile led to a cumulative impact of up to a 2.5-fold increased risk of all-cause mortality and a 2-fold increased risk of MACE. Our findings suggest the importance of considering sleep quality and sleep duration together when developing strategies to improve sleep for adverse cardiovascular events prevention.

Nonetheless, the overwhelming majority of the literature, including some findings from longitudinal studies and meta-analyses, reported a significant association between sleep duration, sleep quality, obstructive sleep apnea, and increased risk of cardiovascular disease in the general population (14, 23). In addition to being a predictor for the progression of primary disease, studies indicate that sleep impairment might also be associated with poor cardiac prognosis after myocardial infarction or CAD (24, 25). A large registry-based study conducted by Clark et al. showed that disturbed sleep in women and impaired awakening in men were associated with a moderately increased risk of adverse cardiac outcomes after a mean follow-up of 10 years (25). The results from our study are consistent with those of previous studies, although different methods were used to evaluate sleep quality, ranging from a self-administered questionnaire, Karolina Sleep Questionnaire, Jenkins Sleep Scale, and other indicators (14, 25, 26).

In an early study, Alcántara et al. reported that individuals with a short duration have a 50% increased risk of myocardial infarction recurrence or mortality in the year after their first AMI event (27). Similarly, results from a more recent study showed that short sleep duration is prevalent and associated with an increased risk of 6-month all-cause readmission (28). Almost 12% of this MINOCA cohort reported an inadequate duration of sleep with fewer than 6 h, which is comparable with the findings of the SOLID-TIMI 52 subgroup analysis of patients after an ACS (29). Our results reveal that the association between short sleep duration and major coronary events in the post-ACS population holds true for patients with MINOCA. Nevertheless, the possible association between prolonged sleep and cardiovascular events has been investigated, conflicting results. In 2019, Krittanawong et al. carried out a meta-analysis on the role of sleep duration and the risk of cardiovascular outcomes, reporting that both short and prolonged sleep duration was associated with a higher risk of overall cardiovascular disease mortality in the general population (30). Likewise, in their study of patients after an ACS, Barger et al. found a U-shaped relationship between nighttime sleep and the risk of cardiac disease, whereby sleeping both too much and too little were associated with an increased risk of cardiovascular mortality (29). However, the MORGEN study demonstrated that long sleep duration tended to be protective for coronary heart disease (14). In the present study, results of outcomes in individuals with a long sleep duration did not reach statistical significance.

We observed in this research that a poorer sleep profile has an additive effect with respect to all-cause mortality and MACE in the MINOCA population. The findings from our trial were consistent with those of epidemiological studies in both healthy individuals and patients with ACS. Previous studies on the relationship between a worsened prognosis and the joint effects of poor sleep quality and short sleep duration in adults aged 40 years or older provided definitive results. The findings of those studies indicated that a lower Sleep Score was associated with an increased risk of CAD in this population (31). Likewise, the combined effect of short sleep duration, obstructive sleep apnea, and shift work history doubled the risk of cardiovascular outcomes in patients with ACS (29). Therefore, when evaluating and improving sleep, both sleep quality and sleep duration should be taken into account.

The exact mechanisms underlying the association between sleep and cardiovascular morbidity have not been completely elucidated. Sleep impairment or short sleep duration was related to decreased leptin and elevated ghrelin levels, which could result in the up-regulation of appetite, elevated food intake, and lower energy expenditure, ultimately facilitating the progression of obesity and impaired glucose tolerance (32). The regulation of other metabolic hormones has also been implicated in sleep disorders and has an association with cardiovascular risks, including increased cortisol secretion, insulin resistance, and altered growth hormone metabolism. In addition, sleep deprivation leads to vascular inflammation, oxidative stress, autonomic nervous system dysregulation, and chronobiological disruption, which impair endothelial function and contribute to the pathogenesis and development of cardiovascular disease (33). Finally, sleep apnea is associated with the increased release of catecholamines and corticotropin-releasing hormone and leads to the activation of the hypothalamic–pituitary–adrenal axis, which may result in metabolic syndrome, elevated blood pressure, and an increased risk of cardiovascular events (34).

The present study has several strengths. First, our study was pragmatic in its design and did not require complex further investigations such as optical coherence tomography imaging, intravascular ultrasonography, or cardiac magnetic resonance with late gadolinium enhancement, and it was oriented toward clinical practice (35). According to the investigator, the above examinations were recommended in selected patients but not mandatory because of insurance infeasibility and poor cost-effectiveness. Thus, the findings of the current study reflect genuine routine clinical practice and are thus applicable to the broad, real-world, MINOCA population (17, 18, 36). Another major strength was the comprehensive collection of data on potential confounders based on our prior knowledge. Sleep disorders are closely related to depression and anxiety, both of which have been found to predict poor prognosis in individuals with CAD and MINOCA. Another major concern is whether PSQI-defined poor sleep quality reflected underlying clinical sleep impairment such as OSAHS, which is common among patients with cardiovascular disease and is a definite predictor for adverse outcomes (19). In addition, impaired sleep was correlated with baseline hypertension, stroke history, and unhealthy lifestyle (e.g., smoking), and although we adjusted our models for all of these confounders, we cannot absolutely exclude that poor sleep might be a risk factor for other underlying disturbances that can affect clinical outcomes in patients with MINOCA. Finally, most patients kept in contact with the help of instant messaging (WeChat), and thus, we achieved a higher quality of follow-up (only five participants were lost to follow-up).

A few limitations of this study should be noted. First, as in most other epidemiological studies, a self-report questionnaire was applied to reflect a general level of sleep complaints within the 1 month prior to initial MINOCA. This may have led to misclassification in terms of sleep assessment at only a single point. However, measures such as polysomnography, which obtain preferable and objective information on sleep, are not feasible in a large population (37). Second, because sleep is a modifiable behavior, sleep quality and duration might be changed in subsequent years, especially in a small portion of patients who agreed to take sleep medication at discharge. Third, this was a single-center study, and the results may not be generalizable to other ethnicities. Fourth, sleep complaints may be largely affected by suffering from an acute attack, such as myocardial infarction. In other words, the validation of the recall of sleeping patterns prior to the initial serious cardiovascular event is a major concern. Fifth, some confounders such as a positive family history of cardiovascular disease and use of medication after discharge were not available and might have influenced our conclusions. Finally, we did not perform intracoronary imaging, cardiac magnetic resonance, and intracoronary provocative testing routinely to assess the specific cause of MINOCA and to exclude MINOCA-mimics (38–40).

## Conclusion

Poor sleep quality is common among Chinese patients with MINOCA. Both poor sleep quality and short sleep duration were independently associated with an increased risk of all-cause mortality and MACE in the MINOCA population. In addition, a poor sleep profile has an additive effect with regard to cardiovascular risks. Thus, efforts should be made to improve both sleep quality and sleep duration in these populations for secondary cardiovascular prevention.

## Data Availability Statement

The raw data supporting the conclusions of this article will be made available by the authors, without undue reservation.

## Ethics Statement

The studies involving human participants were reviewed and approved by the Ethics and Research Committee of the Affiliated Hospital of Jiaxing University. The patients/participants provided their written informed consent to participate in this study.

## Author Contributions

C-YZ: conceptualization, methodology, software, investigation, and writing—original draft. H-LH: data curation, formal analysis, and writing—original draft preparation. G-MT: formal analysis. J-CS: resources, visualization, and investigation. H-XZ: formal analysis, validation, resources, and software. C-LZ: project administration and writing—reviewing and editing. C-JH: conceptualization, methodology, writing—reviewing and editing, and supervision. All authors contributed to the article and approved the submitted version.

## Funding

This research was funded by Jiaxing Science and Technology Program under Grant No. 2020AY30006 and 2021AD30148, Provincial-Municipal Joint Construction of Key Medical Disciplines in Zhejiang Province (2019-ss-xxgbx), Key Discipline Established by Zhejiang Province and Jiaxing City Jointly—Pain Medicine (2019-ss-ttyx), Zhejiang Provincial Health Science and Technology Program under Grant No.2021KY1105, Pioneer innovation team of Jiaxing Arteriosclerotic Diseases Research Institute (XFCX–DMYH), Jiaxing Institute of Arteriosclerotic Diseases (2020-dmzdsys), and Peak Discipline Established by the First Hospital of Jiaxing (GFXK-XXGNK).

## Conflict of Interest

The authors declare that the research was conducted in the absence of any commercial or financial relationships that could be construed as a potential conflict of interest.

## Publisher's Note

All claims expressed in this article are solely those of the authors and do not necessarily represent those of their affiliated organizations, or those of the publisher, the editors and the reviewers. Any product that may be evaluated in this article, or claim that may be made by its manufacturer, is not guaranteed or endorsed by the publisher.
